# Systemic Immune–Inflammatory Markers for Predicting Infarct Volume and Mortality in Patients with Acute Ischemic Stroke: A Retrospective Cohort Study

**DOI:** 10.3390/jcm15124415

**Published:** 2026-06-07

**Authors:** Selim Degirmenci, Erhan Arikan, Mustafa Boz, Ahmet Tugrul Zeytin, Özcan Emre, Efe Sezgin, Mehmet Dokur

**Affiliations:** 1 Department of Emergency Medicine, Faculty of Medicine, Bilecik Şeyh Edebali University, Bilecik 11230, Türkiye; erhan.arikan@bilecik.edu.tr (E.A.); mustafa.boz@bilecik.edu.tr (M.B.); ahmettugrul.zeytin@bilecik.edu.tr (A.T.Z.); mehmet.dokur@bilecik.edu.tr (M.D.); 2 Department of Radiology, Tavşanlı Doç. Dr. Mustafa Kalemli State Hospital, Kutahya 43300, Türkiye; ozcnmre@gmail.com; 3 Department of Molecular Biology and Genetics, İzmir Institute of Technology, Izmir 35430, Türkiye; efesezgin@iyte.edu.tr

**Keywords:** acute ischemic stroke, all-cause mortality, diffusion-weighted magnetic resonance imaging, infarct volume, systemic immune–inflammation index, systemic inflammation response index

## Abstract

**Objectives**: In patients presenting to the emergency department with acute ischemic stroke (AIS), there is a growing need for practical and rapid biomarkers that can effectively predict prognosis and infarct volume, complementing neurological examination and imaging methods. **Methods**: This study retrospectively evaluates patients diagnosed with AIS at the emergency department of Bilecik Training and Research Hospital between 1 March 2022, and 30 September 2023. Patients who were transferred for reperfusion therapy were excluded, as thrombolysis and mechanical thrombectomy were not available at our institution during the study period. The systemic immune–inflammation index (SII) and the systemic inflammation response index (SIRI) scores were calculated based on patients’ laboratory values. Additionally, infarct volumes were independently assessed by two experienced radiologists and calculated by multiplying the hyperintense areas on diffusion-weighted magnetic resonance imaging by slice thickness. **Results**: Of the 349 patients diagnosed with AIS during this study period, 257 who met the inclusion criteria were analyzed. The median age was 76 years (IQR: 66–83), and 130 (51%) patients were female. Lacunar infarction was present in 109 patients (42.4%), while 148 (57.6%) had non-lacunar infarction. The all-cause mortality rates at 1 month, 1 year, and 2 years were 7.8%, 21.4%, and 29.6%, respectively. SII and SIRI scores were significantly higher in deceased patients compared to survivors across all time points for mortality. **Conclusions**: The findings of this study suggest that SII and SIRI values, derived from complete blood count parameters, may contribute to the early identification and management of high-risk AIS patients.

## 1. Introduction

In acute ischemic stroke (AIS), one of the leading causes of mortality and long-term morbidity worldwide, early diagnosis and rapid intervention directly impact patient prognosis. In the emergency department (ED)—the primary point of care for most of these patients—diagnosis is based on neurological examination and imaging, while the severity of the disease is estimated using neurological scoring systems; however, there is a critical need for practical biomarkers that can provide early prognostic insights.

Various studies have shown that immune and inflammatory responses increase following an AIS [1,2]. Although the primary pathology in AIS is ischemia due to vascular occlusion, neuroinflammation is being investigated as a secondary mechanism of damage. Existing evidence suggests that inflammatory factors are associated with both stroke severity and prognosis [3]. Damaged cells activate the immune system when brain tissue remains hypoxic following an AIS [4]. The subsequent inflammatory cytokine storm leads to the release of factors that damage the blood–brain barrier and recruit peripheral immune cells to the lesion site [4]. This triggers the early onset of nerve damage and delays cell repair. Prior studies demonstrate that heightened inflammation subsequent to a stroke can aggravate cerebral damage and deteriorate the prognosis [3]. Therefore, appropriate inflammatory biomarkers may be effective in predicting prognosis in AIS.

The systemic immune–inflammation index (SII) and the systemic inflammation response index (SIRI) are being investigated to predict disease severity and prognosis in conditions characterized by inflammation. Using parameters from a complete blood count (CBC)—a routine test easily performed in EDs—the SII and SIRI reflect the host’s immune and inflammatory states. The SII value is calculated by dividing the product of platelets and neutrophils by the lymphocyte count, and the SIRI is calculated by dividing the product of neutrophils and monocytes by the lymphocyte count. Following evidence that SII is an effective indicator of poor prognosis in patients with hepatocellular carcinoma, its effectiveness has been investigated in numerous conditions, including malignancies, sepsis, diabetes mellitus, ischemic heart disease, and deep vein thrombosis [5,6,7,8,9,10,11]. Although some studies have evaluated mortality in AIS using SII and SIRI, no study has examined their relationship with infarct volume.

The extent of ischemic brain injury can directly influence the magnitude of the systemic inflammatory response, depending on the infarct volume. Larger infarct volumes lead to greater neuronal loss. The release of damage-associated molecular patterns activates peripheral immune cells and strengthens the systemic inflammatory cascade [3]. As a consequence of this mechanism, infarct volume is expected to correlate with composite inflammatory indexes such as SII and SIRI.

The aim of this study is to investigate the relationship between SII and SIRI scores with infarct volume, as well as short-term and long-term mortality, in patients diagnosed with AIS.

## 2. Materials and Methods

### 2.1. Study Design and Population

**Figure 1:**
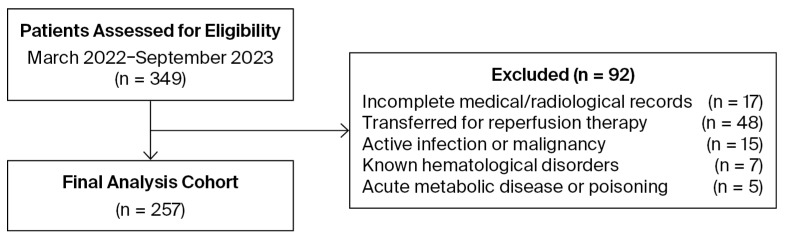
Flow chart outlining patient recruitment and exclusion process. The final analysis cohort comprises 257 out of 349 patients initially assessed for eligibility.

This research was designed as a retrospective cohort study with a level of evidence of 2+ according to the Scottish Intercollegiate Guidelines Network (SIGN 100) criteria [12]. Patients diagnosed with AIS at the ED of Bilecik Training and Research Hospital between 1 March 2022, and 30 September 2023, were retrospectively evaluated. Inclusion criteria were patients aged ≥ 18 years presenting to the ED within 24 h of symptom onset, diagnosed with AIS via diffusion-weighted magnetic resonance imaging (DWI), and with a CBC drawn at presentation. Patients were excluded if their records contained missing information regarding medical history, physical examination, laboratory results, or radiological findings. Additionally, the following exclusion criteria were applied: (i) documented infection before stroke onset or within 72 h of admission; (ii) known hematological disorders; (iii) acute metabolic disease or poisoning; (iv) cancer or current use of steroids or immunosuppressive agents; (v) patients who were transferred to a comprehensive stroke center for potential thrombolytic therapy or mechanical thrombectomy, as these interventions were not available at our facility during the study period; and (vi) patients with intracranial hemorrhage or mass lesions identified on brain computed tomography. The patient recruitment and exclusion process is summarized in the study flow chart (Figure 1).

A sample size of 240 was determined to be sufficient based on the aim of detecting a minimum biomarker difference level of 20% between patients who were alive and those who died, with a two-to-one expected ratio (such as 160 alive and 80 deceased patients). Type I errors (α-level, two-sided) were set to 0.05, and Type II errors (β-level) were set to 0.2 for 80% statistical power.

### 2.2. Data Collection and Indexes

Data were extracted from the hospital information management system, including age, sex, medical history, and blood parameters (white blood cell, platelet, neutrophil, lymphocyte, and monocyte counts), as well as hospital length of stay and mortality dates. Additionally, infarct volumes were calculated based on patients’ DWI images, and SII and SIRI scores were calculated based on laboratory values.

SII and SIRI values were calculated using the CBC performed at hospital admission as follows [5,13]:

$$\mathrm{SII}\;=\;\frac{Neutrophil\times Platelet}{Lymphocyte}$$

$$\mathrm{SIRI}=\frac{Neutrophil\times Monocyte}{Lymphocyte}$$

### 2.3. Imaging and Volumetric Analysis

DWI was performed on a 1.5T scanner (Magnetom Essenza, Siemens, Erlangen, Germany). DWI was acquired using single-shot echo-planar imaging with the following parameters: TE 105 ms, TR 3800 ms, matrix 160 × 160, b-value-1 = 0 and b-value-2 = 1000 s/mm^2^, 5.5 mm slice thickness, 1.65 mm interslice gap, and approximately 20 slices. Two experienced radiologists independently analyzed all DWI data, each blinded to the other’s assessments as well as to clinical and laboratory results, for the presence, location, and number of hyperintensities on DWI. In cases of discordance, consensus was reached through discussion. Lesion volumes were calculated offline using an image post-processing software package (syngo.via, Siemens Healthcare, Erlangen, Germany). The radiologists manually contoured the areas of diffusion hyperintensity on each slice; the total infarct volume was then determined by multiplying the sum of these areas by the slice spacing (slice thickness + interslice gap). Infarcts were categorized as lacunar (<15 mm diameter) or non-lacunar based on the Trial of Org 10172 in Acute Stroke Treatment (TOAST) criteria [14].

### 2.4. Data Analysis

Categorical variables were compared between groups (e.g., lacunar vs. non-lacunar and alive vs. deceased) using Chi-square tests. For continuous variables, normality was assessed using the Shapiro–Wilk test. Non-normally distributed data were analyzed using the Wilcoxon rank-sum test, while Student’s *t*-test was used for normally distributed variables. Univariate and multivariable logistic regression analyses were conducted to identify factors associated with mortality (such as SII and SIRI). Odds ratios (OR) and 95% confidence intervals (CIs) were calculated. Receiver operating characteristic (ROC) analyses were performed based on logistic regression analyses using the pROC package [15]. In order to identify SII and SIRI levels associated with at least a doubled risk of mortality, SII and SIRI values were divided into quartiles, and the thresholds meeting this criterion were determined accordingly. A *p*-value < 0.05 was considered statistically significant. Statistical analyses were conducted using R version 3.6.1 [16].

### 2.5. Ethical Considerations

The study protocol was approved by the Bilecik Şeyh Edebali University Non-Interventional Clinical Research Ethics Committee (Date: 27 November 2024; Approval Number: 2/10), and research was conducted in accordance with the principles of the Declaration of Helsinki.

## 3. Results

### 3.1. Baseline Characteristics

During the study period, 257 out of 349 patients diagnosed with AIS met the inclusion criteria and were included in the final analysis. Female patients accounted for 51% (*n* = 130) of the cohort. The median age was 76 years (IQR: 66–83). Lacunar infarction was present in 109 patients (42.4%), while non-lacunar infarction was observed in 148 patients (57.6%). The most prevalent comorbidities were hypertension (*n* = 74, 29%) and diabetes mellitus (*n* = 42, 16%), whereas newly diagnosed atrial fibrillation was identified in only six patients (2%). The most frequent infarct location was the parietal lobe (*n* = 114, 44%) (Table 1). A total of 172 patients (67%) required hospitalization. The all-cause mortality rates at 1-month, 1-year, and 2-year follow-up were 7.8%, 21.4%, and 29.6%, respectively.

### 3.2. Inflammatory Indexes and Outcomes

A comparison of SII, SIRI, and infarct volume according to sex revealed no significant differences between male and female patients, despite females being significantly older than males (median 79 vs. 73 years, *p* = 0.004) (Table 2). Spearman correlation analyses demonstrated a positive correlation between age and both SII (r = 0.14, *p* = 0.02) and SIRI (r = 0.20, *p* = 0.01), whereas no significant correlation was observed between age and infarct volume (r = 0.03, *p* = 0.66).

**Figure 2:**
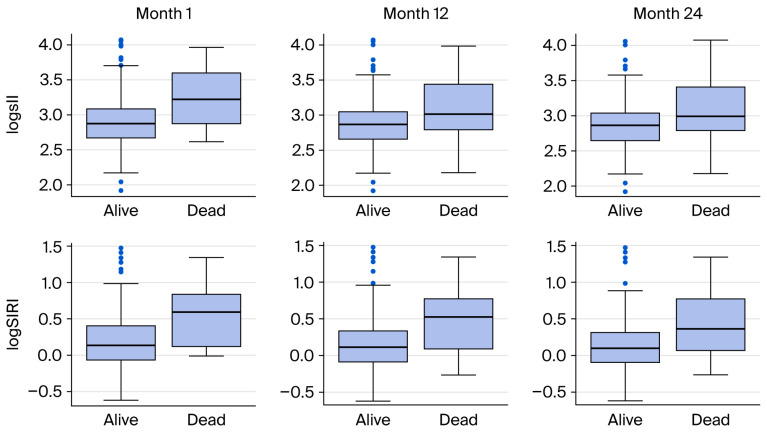
Comparison of SII and SIRI levels between alive and deceased patients at 1, 12, and 24 months.

When comparing survivors and non-survivors, SII scores were significantly higher in deceased patients across 1-month, 1-year, and 2-year time points (*p* = 0.002, *p* = 0.0004, and *p* = 0.0001, respectively). Similarly, SIRI scores were statistically higher in the mortality group (*p* = 0.0003, *p* < 0.0001, and *p* < 0.0001, respectively) (Table 3 and Table 4, Figure 2). Weak but significant positive correlations were observed between infarct volume and both SII (r = 0.14, *p* = 0.02) and SIRI (r = 0.19, *p* = 0.002).

### 3.3. Prognostic Value and Risk Assessment

**Figure 3:**
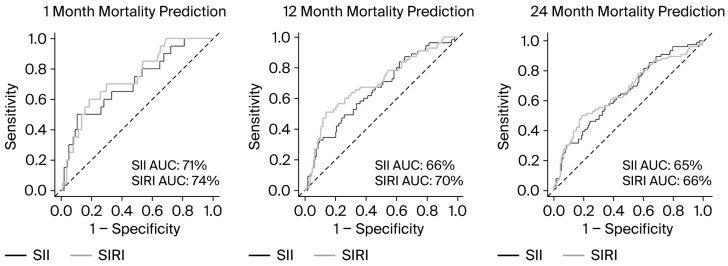
Receiver operating characteristic (ROC) curves of SII and SIRI for predicting mortality at 1, 12, and 24 months. The dashed line represents the reference line (AUC = 0.5).

Increased SII, SIRI, age, and infarct volume were associated with higher mortality risk at all follow-up intervals (Table A1). In multivariable models adjusting for age and infarct volume, elevated SII (OR range: 3.11–4.01) and SIRI (OR range: 3.49–4.40) remained independent predictors of mortality (Table 5). ROC curve analyses further demonstrated the predictive capacity of these indexes. For SII, the AUC (area under the curve) estimates for 1-, 12-, and 24-month mortality were 0.71, 0.66, and 0.65, respectively. For SIRI, the corresponding AUC values were 0.74, 0.70, and 0.66 (Figure 3).

### 3.4. Threshold Analysis

Patients with an SII > 1364 exhibited a 2.54-fold higher risk of 1-month mortality (95% CI: 1.04–6.98, *p* = 0.05), a 2.28-fold risk of 1-year mortality (95% CI: 1.25–4.24, *p* = 0.008), and a 2.23-fold risk of 2-year mortality (95% CI: 1.30–3.87, *p* = 0.004). Similarly, a SIRI score > 2.87 was associated with significantly increased mortality at 1 month (OR: 3.14, 95% CI: 1.21–9.13, *p* = 0.02), 1 year (OR: 3.27, 95% CI: 1.76–6.25, *p* = 0.0002), and 2 years (OR: 2.91, 95% CI: 1.68–5.12, *p* = 0.0002) (Table 6).

## 4. Discussion

In the present study, the relationship between mortality, infarct volume, and inflammatory indexes (SII and SIRI)—calculated from CBC parameters at the time of admission—in patients diagnosed with AIS was evaluated. The findings indicate that both SII and SIRI values are independently associated with all-cause mortality at 1-month, 1-year, and 2-year intervals after adjusting for confounding factors. Furthermore, the positive correlation observed between these indexes and infarct volume suggests a potential link between systemic inflammation and the extent of cerebral tissue damage. These results suggest that rapid, accessible, and cost-effective inflammatory indexes in the ED may serve as valuable tools for predicting prognosis in AIS patients.

Mortality rates among AIS patients vary significantly across studies depending on regional characteristics and healthcare resources. In a large-scale cohort study of over 36,000 AIS patients in Korea, all-cause mortality rates for the post-2015 period were reported as 5.1% at 3 months, 10.1% at 1 year, 14.8% at 2 years, and 20% at 4 years [17]. In a multicenter prospective study analyzing AIS patients in Norway, the 1-year all-cause mortality rate was reported as 7.6% [18]. In another retrospective study conducted in Oman, the mortality rate was reported as 25% at 1 year and 30% at 2 years [19]. The 1-month (7.8%), 1-year (21.4%), and 2-year (29.6%) mortality rates in our study are consistent with the data from Oman [19] but exceed those reported in some Western and East Asian cohorts [17,18]. This discrepancy is attributed to the fact that our study population consisted of patients who did not receive acute reperfusion therapies (thrombolysis or thrombectomy) due to local referral protocols, as well as the advanced mean age of the population in our region.

Huang et al. demonstrated in a cohort of 234 AIS patients that both SII and SIRI scores were significantly higher in those with moderate-to-severe strokes compared to mild cases [20]. Consistent with these findings, recent evidence indicates that SII serves as a reliable predictor for AIS severity, associated complications, and mortality [21,22]. A comprehensive meta-analysis of 78 studies, involving 40,682 participants, further emphasized that elevated SII promotes thrombosis via increased platelet activation and aggregation, thereby exacerbating cerebral ischemia and adversely affecting prognosis [23]. Additionally, a large-scale database analysis of AIS patients admitted to the intensive care unit in Boston revealed that an SIRI value ≥ 4.57 was associated with a significantly higher risk of all-cause mortality at 30 days, 90 days, and 1 year [24]. The findings of the present study largely align with this existing body of literature. The observed ORs (3.11–4.01 for SII and 3.49–4.40 for SIRI) confirm that the prognostic impact of both indexes remains independent of confounding variables. Moreover, the association of elevated SII and SIRI with both short- and long-term mortality suggests that these inflammatory markers may reflect not only neurological damage but also a sustained systemic inflammatory burden and its subsequent complications. In this context, it is also noteworthy that age showed a positive correlation with both SII (r = 0.14; *p* = 0.02) and SIRI (r = 0.20; *p* = 0.01) in the present study. This finding is consistent with the concept of inflammaging—the chronic low-grade systemic inflammatory state associated with advancing age [25]. This association has also been demonstrated in a large-scale 20-year follow-up cohort study of 42,875 US adults, in which elevated SII and SIRI levels were associated with increased all-cause mortality risk, particularly in individuals over 60 years of age. This relationship is attributed to age-related accumulation of senescent cells and their proinflammatory secretory profiles [26]. This age-related inflammatory background may partially contribute to the elevated indexes observed in older patients. Nevertheless, in multivariable logistic regression models adjusting for both age and infarct volume (Table 5), SII and SIRI remained significant independent predictors of mortality across all time points, suggesting that their prognostic utility is not merely a reflection of age-related inflammatory changes but represents an independent contribution to mortality risk.

The prognostic superiority of SIRI in predicting mortality may be attributed to the integration of monocyte counts into the calculation, alongside neutrophils and platelets. Following the activation of the inflammatory cascade, monocytes are known to infiltrate the infarct area and exacerbate secondary brain damage. Conversely, lymphocytes are recognized for their neuroprotective role in immune response following an ischemic insult. Consequently, a high SIRI value—reflecting a synergistic increase in neutrophils and monocytes relative to a decrease in lymphocytes—may provide a more precise representation of the immunological disequilibrium [27]. This pathophysiological rationale potentially explains why the AUC values of SIRI in the present study (0.74 at 1 month, 0.70 at 1 year) were slightly higher than those observed for SII.

A notable finding of this study is the identification of a positive correlation between SII/SIRI and infarct volume. This finding provides a unique perspective, as the majority of existing research has primarily focused on clinical outcomes such as mortality and functional prognosis. Elevated SII levels are hypothesized to facilitate blood–brain barrier disruption by increasing the infiltration of inflammatory cells into the cerebral parenchyma, thereby exacerbating cerebral edema and worsening neurological damage [23]. Based on this mechanism, it is anticipated that larger ischemic lesions trigger a more robust systemic inflammatory response, resulting in higher SII and SIRI values [28]. Although the correlations observed in the present study (r = 0.14 for SII and r = 0.19 for SIRI) were statistically significant, their modest magnitude suggests that these indexes should not be regarded as surrogate markers of infarct size. Rather, this association may reflect a bidirectional relationship between cerebral tissue damage and systemic inflammation and should be interpreted as an exploratory finding that requires validation in larger prospective studies.

From a clinical perspective, the rapid availability of SII and SIRI through routine CBC analysis highlights their practical utility in the ED setting. Although the National Institutes of Health Stroke Scale (NIHSS) and advanced imaging remain the gold standard prognostic tools in AIS, clinicians often benefit from additional objective markers during the initial hours of assessment. In this context, integrating SII and SIRI into routine clinical protocols could facilitate the rapid identification of high-risk patients, particularly in the hyperacute phase when timely intervention is paramount. Although the AUC values obtained in the present study reflect modest discrimination when these indexes are used as standalone predictors, their primary prognostic utility is their independent contribution within multivariable models. When adjusted for age and infarct volume, both SII and SIRI remained significant independent predictors of mortality across all time points, suggesting that they provide incremental prognostic value beyond established clinical variables.

Based on the threshold values identified in this study, patients with an SII above 1364 or SIRI above 2.87 may be considered at high risk for short- and long-term mortality. When combined with established prognostic variables such as age, NIHSS score, and infarct volume, these indexes may contribute to a more comprehensive and accurate prognostic assessment in AIS patients. In clinical practice, such high-risk patients may benefit from closer monitoring in the ED, early intensive care unit evaluation, and more aggressive secondary prevention strategies.

The present study was conducted in a setting where reperfusion therapy was not available, and patients transferred for thrombolysis or mechanical thrombectomy were excluded. While this introduces a selection bias and limits the generalizability of our findings to the broader AIS population, it is noteworthy that the cohort studied reflects a clinically significant and globally prevalent patient group. Reperfusion therapy remains inaccessible or underutilized in a substantial proportion of AIS cases worldwide, particularly in non-comprehensive stroke centers, low- and middle-income countries, and among patients presenting beyond the therapeutic time window for acute interventions. In these settings, where advanced interventional options are unavailable, SII and SIRI may hold particular clinical value in identifying high-risk patients and guiding early management decisions. Future studies conducted in comprehensive stroke centers should investigate the prognostic utility of SII and SIRI in patients who receive reperfusion therapy. This would enable a more complete characterization of indexes across the full spectrum of AIS management.

Several limitations of the present study should be acknowledged. First, its retrospective and single-center design may limit the generalizability of the findings to broader populations. Second, SII and SIRI were calculated only at the time of admission; therefore, the impact of dynamic inflammatory fluctuations throughout the clinical course could not be assessed. Third, the use of DWI as the gold standard for diagnosis resulted in the exclusion of patients who could not undergo DWI or those with DWI-negative results. Furthermore, as discussed above, our findings are specifically representative of a population that did not receive acute reperfusion therapy. Finally, NIHSS scores were not routinely documented in the hospital records during the study period and could not be retrieved retrospectively, which precluded their inclusion in the multivariable models. As NIHSS is a primary determinant of both stroke severity and prognosis, its absence represents a significant limitation, and residual confounding cannot be fully excluded. Similarly, stroke etiology could not be systematically incorporated due to inherent missing data in retrospective records. Furthermore, the threshold values reported in Table 6 are dataset-specific and may not be directly generalizable to other populations; external validation in independent cohorts is therefore warranted. Despite these constraints, this study remains one of the few to examine the direct association between systemic inflammatory indexes and infarct volume, providing a foundation for future multicenter prospective trials.

## 5. Conclusions

In conclusion, SII and SIRI scores based on standard CBC parameters serve as independent prognostic markers of short- and long-term mortality in patients with AIS. The modest but statistically significant correlation between these indexes and infarct volume suggests a potential link with the systemic inflammatory response associated with cerebral tissue damage; however, this finding is exploratory and should be interpreted with caution. Nevertheless, given their independent association with mortality, incorporating these markers into clinical practice alongside other diagnostic indicators could facilitate the early identification of high-risk patients and improve management strategies. Further multicenter, large-scale clinical trials are warranted to validate these results and expand their clinical utility.

## Figures and Tables

**Table 1:** Baseline characteristics and mortality rates of patients according to infarct type.

Variable	Lacunar (*n* = 109)	Non-Lacunar (*n* = 148)	Total (*n* = 257)
Age (year)	75 (65–83)	76 (67–83)	76 (66–83)
Sex, Female	51 (39%)	79 (61%)	130 (51%)
Medical History			
Hypertension	34 (46%)	40 (54%)	74 (29%)
Diabetes mellitus	19 (45%)	23 (55%)	42 (16%)
Hyperlipidemia	3 (75%)	1 (25%)	4 (2%)
Chronic renal failure	2 (67%)	1 (33%)	3 (1%)
Coronary artery disease	2 (33%)	4 (67%)	6 (2%)
Congestive heart failure	3 (43%)	4 (57%)	7 (3%)
Previous AIS	21 (34%)	40 (66%)	61 (24%)
Previous AF	2 (29%)	5 (71%)	7 (3%)
Newly diagnosed AF	1 (17%)	5 (83%)	6 (2%)
Infarct side			
Left	54 (45%)	67 (55%)	121 (47%)
Right	47 (41%)	68 (59%)	115 (45%)
Bilateral	8 (38%)	13 (62%)	21 (8%)
Infarct zone			
Parietal	39 (34%)	75 (66%)	114 (44%)
Occipital	10 (15%)	55 (85%)	65 (25%)
Frontal	15 (37%)	26 (63%)	41 (16%)
Thalamus	24 (63%)	14 (37%)	38 (15%)
Cerebellum	13 (41%)	19 (59%)	32 (12%)
Temporal	9 (31%)	20 (69%)	29 (11%)
Pons	11 (85%)	2 (15%)	13 (5%)
Infarct volume (cm^3^)	0.49 (0.29–0.97)	5.77 (2.67–17.53)	1.83 (0.59–8.07)
Hospital admission	61 (56%)	111 (75%)	172 (67%)
Length of hospital stay (day)	1 (0–3)	2 (1–4)	2 (0–4)
1-month mortality	3 (2.8%)	17 (11.5%)	20 (7.8%)
1-year mortality	17 (15.6%)	38 (25.7%)	55 (21.4%)
2-year mortality	25 (22.9%)	51 (34.5%)	76 (29.6%)

Notes: Data reported as number (%) or median (IQR). Statistical significance was defined as *p* < 0.05. Abbreviations: AIS, acute ischemic stroke; AF, atrial fibrillation.

**Table 2:** Comparison of age, SII, and SIRI between males and females.

Variable	Male (*n* = 127)	Female (*n* = 130)	*p*
Age	73 (65–81)	79 (69–84)	0.004
Indexes			
SII	764 (458–1515)	781 (497–1248)	NS
SIRI	1.68 (0.97–2.88)	1.30 (0.85–2.51)	NS
Infarct volume	1.62 (0.54–8.71)	2.04 (0.69–5.62)	NS

Notes: Data were reported as the median (IQR). Statistical significance was defined as *p* < 0.05. Abbreviations: SII, systemic immune–inflammation index; SIRI, systemic inflammatory response index; NS, not significant (*p* ≥ 0.05).

**Table 3:** Comparison of demographic, clinical, and laboratory variables between alive and deceased patients at 1 month.

Variable	1-Month Mortality		
	Alive (*n* = 237)	Dead (*n* = 20)	*p*
Sex, Female	119 (92%)	11 (8%)	NS
Age (year)	74 (65–83)	83.5 (80–88.5)	0.0001
Laboratory			
WBC	7.9 (6.9–9.9)	9.4 (8.3–13)	0.01
PLT	234 (190–278)	238 (153–279)	NS
Lymphocyte	1.7 (1.3–1.8)	0.9 (0.6–1.4)	0.02
Neutrophil	5.4 (4.2–6.3)	7.7 (6.1–9.4)	0.002
Monocyte	0.47 (0.36–0.49)	0.52 (0.38–0.53)	NS
Indexes			
SII	750 (468–1218)	1763 (754–3880)	0.002
SIRI	1.4 (0.9–2.5)	3.9 (1.3–6.5)	0.0003
Hospital admission	155 (65%)	17 (85%)	NS
Length of hospital stay (day)	2 (0–4)	7 (1–18)	0.005
Infarct type			
Lacunar	106 (45%)	3 (15%)	0.01
Non-lacunar	131 (55%)	17 (85%)	
Infarct volume (cm^3^)	1.6 (0.5–5.6)	14.2 (9–31.7)	<0.0001

Notes: Data were reported as number (%) or median (IQR). Statistical significance was defined as *p* < 0.05. Abbreviations: WBC, white blood cell; PLT, platelet; SII, systemic immune–inflammation index; SIRI, systemic inflammatory response index; NS, not significant (*p* ≥ 0.05).

**Table 4:** Comparison of demographic, clinical, and laboratory variables between alive and deceased patients at 1 and 2 years.

	12-Month Mortality			24-Month Mortality		
Variable	Alive (*n* = 202)	Dead (*n* = 55)	*p*	Alive (*n* = 181)	Dead (*n* = 76)	*p*
Sex, Female	98 (75%)	32 (25%)	NS	90 (69%)	40 (31%)	NS
Age (year)	73.5 (64.3–82)	82 (74–86.5)	<0.0001	73 (64–82)	81 (74–85.3)	<0.0001
Laboratory						
WBC	7.8 (6.8–9.6)	9.2 (7.7–12.8)	0.0007	7.7 (6.7–9.6)	9 (7.6–10.5)	0.0003
PLT	233 (190–279)	238 (171–275)	NS	232 (192–279)	238 (175–277)	NS
Lymphocyte	1.7 (1.3–1.9)	1.4 (0.8–1.5)	0.01	1.8 (1.3–1.9)	1.4 (0.9–1.5)	0.003
Neutrophil	5.2 (4.1–6.1)	6.8 (4.9–8.3)	<0.0001	5.1 (4.1–5.9)	6.5 (4.9–8.2)	<0.0001
Monocyte	0.46 (0.36–0.48)	0.51 (0.42–0.55)	0.02	0.46 (0.36–0.47)	0.53 (0.40–0.54)	0.004
Indexes						
SII	730 (455–1112)	1023 (617–2750)	0.0004	726 (439–1078)	984 (614–2494)	0.0001
SIRI	1.3 (0.8–2.2)	3.4 (1.2–5.9)	<0.0001	1.25 (0.80–2.05)	2.31 (1.18–5.87)	<0.0001
Infarct Type						
Lacunar	92 (46%)	17 (31%)	NS	84 (46%)	25 (33%)	0.045
Non-lacunar	110 (54%)	38 (69%)		97 (54%)	51 (67%)	
Infarct volume (cm^3^)	1.4 (0.5–4.8)	8.4 (1.1–24.2)	<0.0001	1.30 (0.49–4.55)	5.87 (1.08–22.12)	<0.0001
Hospital admission	124 (61%)	48 (87%)	0.0003	110 (61%)	62 (82%)	0.001
LOS (day)	1.5 (0–3)	7 (1–21)	<0.0001	1 (0–3)	3.5 (1–19)	<0.0001

Notes: Data are reported as number (%) or median (IQR). Statistical significance was defined as *p* < 0.05. Abbreviations: WBC, white blood cell; PLT, platelet; SII, systemic immune–inflammation index; SIRI, systemic inflammatory response index; LOS, Length of hospital stay; NS, not significant (*p* ≥ 0.05).

**Table 5:** Multivariable logistic regression analyses examining the relationship between SII, SIRI, and mortality, adjusting for age and infarct volume.

	1-Month Mortality		12-Month Mortality		24-Month Mortality	
Variable	OR_adj_ (95%)	P_adj_	OR_adj_ (95%)	P_adj_	OR_adj_ (95%)	P_adj_
SII	4.01 (1.09–15.5)	0.04	3.11 (1.29–7.72)	0.01	3.49 (1.60–7.87)	0.002
SIRI	3.49 (0.9–13.2)	0.006	3.74 (1.59–9.06)	0.003	4.40 (2.05–9.74)	0.0002

All models are adjusted for age and infarct volume.

**Table 6:** Odds ratios for mortality according to SII and SIRI threshold groups at 1, 12, and 24 months.

Variable	1-Month Mortality		12-Month Mortality		24-Month Mortality	
	OR (95%)	*p*	OR (95%)	*p*	OR (95%)	*p*
**SII**						
Group 1 (≤1364)	Ref	Ref	Ref	Ref	Ref	Ref
Group 2 (>1364)	2.5 (1.0–7.0)	0.05	2.3 (1.3–4.2)	0.008	2.2 (1.3–3.9)	0.004
**SIRI**						
Group 1 (≤2.87)	Ref	Ref	Ref	Ref	Ref	Ref
Group 2 (>2.87)	3.1 (1.2–9.1)	0.02	3.3 (1.8–6.3)	0.0002	2.9 (1.7–5.1)	0.0002

## Data Availability

The data of this study are available from the corresponding author upon reasonable request.

## Abbreviations

The following abbreviations are used in this manuscript:

AIS	Acute ischemic stroke
AUC	Area under the curve
CBC	Complete blood count
CI	Confidence interval
DWI	Diffusion-weighted magnetic resonance imaging
ED	Emergency department
IQR	Interquartile range
NIHSS	National Institutes of Health Stroke Scale
OR	Odds ratio
ROC	Receiver operating characteristic
SII	Systemic immune–inflammation index
SIRI	Systemic inflammation response index

## Appendix A

**Table A1:** Logistic regression analyses examining the relationship between SII, SIRI, infarct volume measurements, and mortality at 1, 12, and 24 months.

	1-Month Mortality		12-Month Mortality		24-Month Mortality	
Variable	OR (95%)	*p*	OR (95%)	*p*	OR (95%)	*p*
Age	1.11 (1.05–1.19)	0.0006	1.09 (1.05–1.13)	0.00001	1.06 (1.03–1.10)	0.00002
SII	6.98 (2.35–21.51)	0.0005	4.16 (1.94–9.23)	0.0003	4.35 (2.13–9.26)	0.00001
SIRI	6.62 (2.38–19.07)	0.0003	5.33 (2.55–11.57)	0.00001	5.91 (2.93–12.53)	0.00001
Infarct volume (cm^3^)	4.39 (2.29–9.14)	0.0001	2.58 (1.71–3.99)	0.00001	2.22 (1.54–3.29)	0.0003
